# The role of transcriptional ‘futile cycles’ in autophagy and microbial pathogenesis

**DOI:** 10.15698/mic2015.08.221

**Published:** 2015-07-30

**Authors:** Guowu Hu, Travis McQuiston, Amélie Bernard, Yoon-Dong Park, Jin Qiu, Ali Vural, Nannan Zhang, Scott R. Waterman, Nathan H. Blewett, Timothy G. Myers, John H. Kehrl, Gulbu Uzel, Daniel J. Klionsky, Peter R. Williamson

**Affiliations:** 1 Laboratory of Clinical Infectious Diseases, National Institute of Allergy and Infectious Diseases, National Institutes of Health, Bethesda, MD, USA, 20892.; 2 Life Sciences Institute, University of Michigan, Ann Arbor, MI, USA 48109.; 3 Laboratory of Immunoregulation, National Institute of Allergy and Infectious Diseases, National Institutes of Health, Bethesda, MD, USA, 20892.; 4 Intramural Research Program in Genomics of Differentiation, National Institute of Child Health and Human Development, National Institutes of Health, Bethesda, MD 20892.; 5 Genomic Technologies Section, Research Technologies Branch, National Institute of Allergy and Infectious Diseases, National Institutes of Health, MD, USA, 20892.

**Keywords:** autoimmunity, autophagy, fungus, pathogen, phosphorylation, TOR, translation, virulence

## Abstract

Eukaryotic cells utilize macroautophagy (hereafter autophagy) to recycle cellular materials during nutrient stress. Target of rapamycin (Tor) is a central regulator of this process, acting by post-translational mechanisms, phosphorylating preformed autophagy-related (Atg) proteins to repress autophagy during log-phase growth. We recently reported an additional role for post-transcriptional regulation of autophagy, whereby the mRNA decapping protein, Dcp2, undergoes Tor-dependent phosphorylation, resulting in increased *ATG* mRNA decapping and degradation under nutrient-rich, repressing conditions. Dephosphorylation of Dcp2 during starvation is associated with dissociation of the decapping-*ATG* mRNA complex, with resultant stabilization of, and accumulation of, *ATG* transcripts, leading to induction of autophagy. Regulation of mRNA degradation occurs in concert with known mRNA synthetic inductive mechanisms to potentiate overall transcriptional regulation. This mRNA degradative pathway thus constitutes a type of transcriptional ‘futile cycle’ where under nutrient-rich conditions transcript is constantly being generated and degraded. As nutrient levels decline, steady state mRNA levels are increased by both inhibition of degradation as well as increased *de novo* synthesis. A role for this regulatory process in fungal virulence was further demonstrated by showing that overexpression of the Dcp2-associated mRNA-binding protein Vad1 in the AIDS-associated pathogen *Cryptococcus neoformans* results in constitutive repression of autophagy even under starvation conditions as well as attenuated virulence in a mouse model. In summary, Tor-dependent post-transcriptional regulation of autophagy plays a key role in the facilitation of microbial pathogenesis.

Adaptation to the hostile host environment is an important component of pathogenic fitness and survival. *Cryptococcus neoformans* is a major fungal pathogen that causes death in an estimated half a million patients annually. Metabolic, transcriptional and gene deletion studies have demonstrated that an important attribute of cryptococcal pathogenesis is its ability to rapidly adapt and thrive in nutrient-poor host niches such as the macrophage phagolysosome and within brain tissue. Induction of high affinity glucose transporters and genes involved in gluconeogenesis as well as pro-survival mechanisms such as autophagy allow survival and effective host damage by the fungus. Autophagy, for example, enables cellular recycling during nutrient deprivation to provide essential building blocks for metabolic processes. Such metabolic reprogramming that allows for the success of *C. neoformans* in low-nutrient environments may also explain why drug resistance testing that relies on growth inhibition under high-nutrient conditions may be poorly predictive of therapeutic failure for this organism. For example, agents such as fluconazole that exhibit low mean inhibitory concentrations in media containing glucose, are poorly effective as an initial therapy against cryptococcal clinical infections in standard doses. Instead, fungicidal therapies that kill, independent of substrate concentrations, such as amphotericin B, are more effective both for fungal clearance and clinical response. Thus, an understanding of cellular mechanisms facilitating low-nutrient survival and host damage is important to the study of cryptococcal pathogenesis.

Regulation of autophagy must both prevent unnecessary cellular damage during growth and allow rapid induction during stress or nutrient deprivation. Tor is an important post-translational repressor of autophagy, phosphorylating constitutive autophagy-related proteins such as Atg13 when nutrients are available, thereby inhibiting autophagic induction. Although transcription-dependent regulation occurs in both yeast and mammalian cells, less is known about post-transcriptional regulation and how it might serve to potentiate these other mechanisms. Recent work has suggested that mRNA stability may be accentuated in response to Tor-dependent signals by the Rim15 pathway, acting in synergy with transcriptional and post-translational mechanisms. However, mechanisms that regulate mRNA degradation have remained obscure.

Whereas the regulatory capacity of mRNA degradation has been poorly understood, mechanistic steps leading to deadenylation-dependent mRNA degradation in eukaryotes are well known. This has been most studied with regards to anabolic genes that are highly expressed under mid-log phase growth but require suppression during the transition to low growth states as substrates are used up. Deadenylation is thought to be a reversible step, followed by irreversible removal of the mRNA 5’ cap by the decapping enzyme Dcp2, and subsequent degradation in a 5’-3’ direction by the exoribonuclease, Xrn1. In addition, a family of ancillary proteins include an RCK member of RNA-binding DExD/H-box proteins—Dhh1 in *Saccharomyces cerevisiae*, Vad1 in the fungal pathogen *C. neoformans*. *In vitro *data have suggested that mRNA decay is intimately associated with mRNA translational arrest that often precedes mRNA decay and occurs in puncta referred to as processing (P)-bodies. However, an important difference between anabolic genes and autophagy-related (*ATG*) genes are that those related to autophagy are repressed during log-phase growth and induced during nutrient starvation—the exact opposite of anabolic genes involved in log-phase growth that are repressed during starvation. Thus, *ATG* genes may require subtle differences in induction programs from those previously studied.

Independent studies of the RCK proteins Dhh1 in *S. cerevisiae* and Vad1 in *C. neoformans* in our laboratories suggested a role for post-transcriptional control of autophagy. Screening a library of RNA-binding protein mutants of *S. cerevisiae* for a cell survival phenotype identified Dhh1 as a potential regulator, and immunoprecipitation of Vad1 from *C. neoformans* identified *ATG8* mRNA bound to the decapping complex. Mutational studies in both *S. cerevisiae* and *C. neoformans* found that Dhh1 and Vad1, respectively, suppress steady state levels of multiple *ATG* mRNAs and inhibit autophagic flux. In *C. neoformans*, the initial data were extended to show that recruitment of *ATG* mRNA to the RCK/Vad1 complex is a dynamic process whereby *ATG* mRNA is recruited for decapping and degradation during nutrient-replete conditions and released during starvation to provide increased mRNA steady state levels for polysome-dependent translation. Previous studies demonstrate binding of yeast RCK/Dhh1 to the Dcp2-containing decapping complex, suggesting a role for RCK members in mRNA recruitment to this complex. Additional studies using a PCR-based mRNA decapping assay and time-course studies of transcriptional degradation after mRNA synthesis inhibition using 1,10-phenathroline suggest that recruitment to the RCK/Vad1 complex is accompanied by increased *ATG* mRNA degradation, and mRNA release is accompanied by transcript stabilization and increased protein translation. Previous studies have demonstrated the role of mRNA stabilization by Rim15-dependent mechanisms including proteins such as Igo1/2 that could provide mRNA stabilization during starvation. Additional studies show that these changes in *ATG* mRNA decapping are mediated by Tor-dependent phosphorylation of Dcp2, which facilitates mRNA recruitment to the decapping complex, followed by decapping and degradation. In summary, the data suggest that autophagy repression is a product of basal *ATG* mRNA synthesis (perhaps induced by transcription factors such as Gln3 in yeast and TFEB in mammals) and matched degradation in a type of transcriptional futile cycle.

Futile cycles are well known in metabolism such as that involved in phosphorylation and dephosphorylation of fructose 6-phosphate and fructose 1,6-bisphosphate (Fig. 1) where substrates undergo rapid interconversion at equal rates in the resting state. During the ‘flight or fight’ response, gluconeogenesis is triggered in muscle cells, where high levels of fructose 1,6-biphosphate are rapidly mobilized. The existence of such energy intensive cycles was initially quite puzzling because of the obvious excessive energy consumed by such a cycle during the resting state. However, such a cycle retains high levels of phosphorylation/dephosphorylation-dependent enzyme activities that would be difficult to assemble *de novo* in a timely fashion. Rapid synthesis of fructose 1,6-bisphosphate is facilitated by the constitutive high levels of fructose 6-phosphate phosphorylation, and requires only small increases in rates of phosphorylation and small decreases in rates of dephosphorylation to produce large increases in fructose 1,6-bisphosphate for gluconeogenesis. In the same way (Fig. 1), maintenance of both mRNA synthesis and degradation of *ATG* genes during log-phase growth provides a more rapid inductive program during the transition to starvation where small changes in mRNA synthesis, accompanied by the inhibition of Tor-dependent *ATG* transcript degradation, results in relatively larger increases in mRNA steady state levels. Pathogen survival is thus facilitated by transcriptional futile cycles, which bears similarity to post-translational mechanisms by optimizing constitutively active synthetic machinery to rapidly induce autophagy during transition to the hostile host environment.

**Figure 1:**
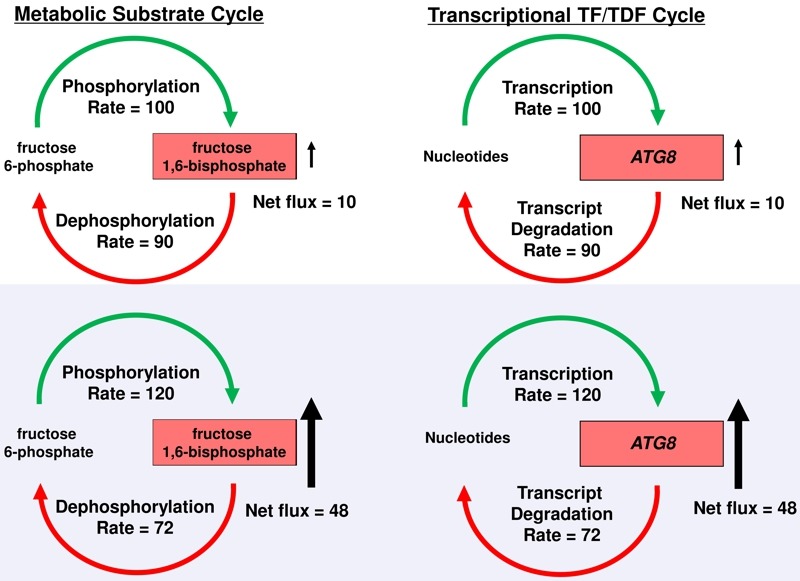
FIGURE 1: Efficiency of metabolic and transcriptional “futile cycles”. Top panels illustrate repressive conditions for the generation of fructose 1,6-bisphosphate (resting muscle) and ATG8 transcripts (nutrient-replete conditions), respectively. Lower panels illustrate the corresponding inductive conditions, active muscle contraction and starvation, respectively. This figure was modified from figure 7 of Park et al., (2010) J. Biol. Chem. 285:34746.

